# Diffusion-augmented contrastive learning for diabetic retinopathy severity classification

**DOI:** 10.3389/frai.2026.1932259

**Published:** 2026-10-12

**Authors:** Ali Abidalkareem, Ali K. Ibrahim, Hanqi Zhuang

**Affiliations:** Department of Electrical Engineering and Computer Science, Florida Atlantic University, Boca Raton, FL, United States

**Keywords:** class imbalance, contrastive learning, convolutional neural networks, denoising diffusion probabilistic models, diabetic retinopathy, domain shift, ensemble learning, external validation

## Abstract

Automated diabetic retinopathy (DR) severity classification faces persistent challenges from limited annotated datasets, severe class imbalance across the five-level International Clinical Diabetic Retinopathy (ICDR) scale, and domain shift between imaging environments. This paper presents an integrated deep learning framework that extends our earlier Fused Ensemble Convolutional Inception Network (93.2% accuracy) through three complementary innovations. First, a class-conditional latent denoising diffusion probabilistic model (DDPM) generates anatomically realistic synthetic fundus images for underrepresented DR severity levels, addressing data scarcity and class imbalance simultaneously. Second, a SimCLR contrastive self-supervised pre-training protocol, applied to the combined real-and-synthetic corpus, learns discriminative retinal representations prior to supervised fine-tuning. Third, a five-model ensemble (EfficientNet-B3, ResNet-50, VGG16, VGG19, and DenseNet-121) fused via a learned Pattern-Net achieves improved generalization through architectural diversity. Evaluated on the APTOS 2019 dataset under 5-fold stratified cross-validation, the integrated framework achieves 95.1% accuracy, 0.928 quadratic weighted kappa (QWK), 0.91 F1-score, and 0.97 AUC. Class-wise improvements over the EfficientNet-B3 baseline reach +23.8 percentage points for Severe DR (61.8 → 85.6%) and +17.7 for Mild DR (71.2 → 88.9%). Independent zero-shot validation on EyePACS yields 78.4% accuracy, 0.809 QWK, 0.738 macro F1, and 0.887 macro AUC, quantifying the expected performance reduction under domain shift. Fold-level variability and paired out-of-fold statistical analyses are additionally reported, and Grad-CAM visualization is included for qualitative model interpretability. These results demonstrate the synergistic benefit of generative augmentation with self-supervised representation learning for imbalanced medical image classification, with implications for scalable automated DR screening in resource-limited settings.

## 1 Introduction

Diabetic retinopathy (DR) is the leading cause of preventable blindness among working-age adults worldwide, affecting an estimated 103 million individuals in 2020, a figure projected to reach 160 million by 2045 (Teo et al., 2021). The disease progresses asymptomatically through well-defined severity stages, from the earliest microaneurysm formation through progressive hemorrhages, venous beading, and intraretinal microvascular abnormalities, ultimately culminating in sight-threatening neovascularization (Wilkinson et al., 2003). Timely detection at moderate or severe non-proliferative stages can prevent up to 98% of severe vision loss (Scanlon, 2017), yet specialist ophthalmologist access remains severely constrained in resource-limited settings.

Convolutional neural networks (CNNs) have transformed automated DR grading since Gulshan et al. (2016) demonstrated ophthalmologist-level sensitivity and specificity. Subsequent architectures including DenseNet, EfficientNet, and hybrid CNN-Transformer models have progressively improved benchmark performance (Macsik et al., 2024; Khandait, 2026; Zhou et al., 2023). However, three fundamental barriers persist: (i) scarcity of expert-annotated fundus images, particularly for rare severe and proliferative stages; (ii) severe class imbalance in DR datasets, such as APTOS 2019, where the No-DR class contains 1,805 images (49.3%) compared with only 193 Severe-DR images (5.3%), a greater than nine-fold difference; and (iii) performance degradation from domain shift across imaging systems and patient cohorts.

In our earlier work (Abidalkareem et al., 2020), we introduced the *Fused Ensemble Convolutional Inception Network* (FECIN), which achieved 93.2% accuracy on five-class DR severity classification by randomly generating and fusing an ensemble of inception-based CNNs. Although FECIN demonstrated that model diversity through random architecture generation improves ensemble generalization, it relied exclusively on geometric data augmentation and was limited to 100 × 100 resolution inputs.

This paper presents a substantially extended framework addressing these limitations through three integrated innovations. First, class-conditional DDPMs (Ho et al., 2020) synthesize high-fidelity fundus images for underrepresented severity levels. Second, SimCLR contrastive self-supervised learning Chen et al. (2020) pre-trains an encoder on the combined real-and-synthetic corpus, learning discriminative retinal representations without label supervision. Third, the ensemble is expanded to include architecturally diverse pre-trained models fused by a learned Pattern-Net (Li et al., 2018). The main contributions of this work are seven-fold. First, we introduce a class-conditional latent DDPM pipeline capable of generating anatomically realistic fundus images, whose visual plausibility is assessed using FID analysis and an ophthalmologist realism review rather than claimed as definitive lesion-level clinical validation. Second, we develop a fundus-adapted SimCLR pre-training strategy that incorporates synthetic images to improve encoder representations for diabetic retinopathy classification. Third, we propose a multi-architecture ensemble with learned fusion, achieving a 5.3 % improvement over the best single baseline, with additional gains from contrastive pre-training (+1.1 %) and diffusion-based augmentation (+0.8 %). Fourth, we provide a comprehensive ablation study that quantifies the contribution of each component and highlights clinically meaningful improvements for minority classes in DR screening. Fifth, we add an independent EyePACS zero-shot validation protocol to quantify generalization under domain shift. Sixth, we expand statistical reporting with fold-level variability, confidence intervals, McNemar testing for paired classification accuracy, and paired bootstrap analysis of QWK, macro F1, and macro AUC using out-of-fold predictions. Seventh, we add Grad-CAM activation visualization across all five DR grades as a qualitative interpretability aid.

## 2 Related work

### 2.1 Automated DR classification

Deep CNN approaches have rapidly advanced automated DR grading. Pratt et al. (2016) trained a custom CNN on 80,000 Kaggle EyePACS images, achieving 75% five-class accuracy. Transfer learning became standard practice: Nair et al. (2022) reported ResNet-50 at 97.2% on APTOS 2019, while Mohanty et al. (2023) achieved 97.3% with DenseNet-121. Bilal et al. (2022) reached 97.92% on EyePACS-1 using a hybrid dual U-Net segmentation plus Inception-V3 pipeline. Recent APTOS-focused work also includes EfficientNet ensembles, vision transformers, and retina foundation models, as summarized with protocol-specific metrics in Section 2.4.

### 2.2 Data augmentation and synthetic generation

İncir and Bozkurt (2024) showed affine augmentation on EyePACS raised EfficientNetV2-M accuracy from 78.61% to 97.65%. GAN-based fundus synthesis has been explored Costa et al. (2018) but suffers from training instability. Denoising diffusion probabilistic models have emerged as superior alternatives: Go et al. (2024) demonstrated structural realism in diffusion-generated fundus images; Feng et al. (2024) proposed a two-stage conditional diffusion framework for DR lesion synthesis; Nderitu et al. (2025) showed that conditional cascaded diffusion models improve downstream DR prognosis prediction; and Patti et al. (2025) validated diffusion-based counterfactual fundus generation as clinically plausible.

### 2.3 Self-supervised and contrastive learning

SimCLR (Chen et al., 2020) learns representations by maximizing agreement between augmented views of the same image using the Normalized Temperature-scaled Cross-Entropy (NT-Xent) loss. MoCo (He et al., 2020) extends this with a dynamic queue of negative keys, while BYOL (Grill et al., 2020) eliminates negative samples through an asymmetric online-target network. In ophthalmology, Huang et al. (2021) demonstrated lesion-guided contrastive learning for DR grading, and Multi-Instance Contrastive Learning (Azizi et al., 2021) constructs positive pairs from images of the same patient, capturing clinically meaningful patient-level features.

### 2.4 Vision transformers, foundation models, and recent self-supervised approaches

Recent DR research has expanded beyond conventional CNN ensembles toward vision transformers, retina-specific foundation models, and self-supervised pre-training. Khandait (2026) compared ViT-Base/16, Swin Transformer, and InceptionResNetV2 on the five-class APTOS 2019 task, reporting 85.40% accuracy and macro F1 of 0.7247 for ViT-Base/16 and 83.20% accuracy for Swin Transformer. Zhou et al. (2023), a retina-specific foundation model pre-trained using masked autoencoding on a large retinal corpus, reported an AUROC of 0.943 on APTOS 2019 after fine-tuning. Ohri et al. (2024) evaluated DINO, masked autoencoder (MAE), and masked Siamese network pre-training with a vision transformer on EyePACS, with the MAE-based model reporting a kappa score of 0.9027 in the full-data regime. Macsik et al. (2024) reported an EfficientNetB4 ensemble on APTOS 2019 with mean accuracy of 89.90% and quadratic weighted kappa of 0.917 under their 80/20 evaluation protocol.

These studies demonstrate the breadth of current DR modeling strategies, but their reported values should not be treated as a strict ranking against the present framework because the data splits, pre-processing pipelines, training regimes, and reported metrics differ across studies. The comparison is therefore used to position the proposed method within the current literature rather than to claim a controlled head-to-head advantage.

### 2.5 Cross-dataset generalization in DR screening

Domain shift across fundus camera hardware, illumination protocols, and population demographics is a well-documented limitation for DR classifiers trained on a single source dataset. Studies have reported accuracy degradation when models trained on one fundus corpus are evaluated on another. This motivates the independent EyePACS external-validation protocol introduced in Section 3.4.

## 3 Methodology

### 3.1 Datasets

Two publicly available retinal fundus image datasets, EyePACS and APTOS 2019 (Aravind Eye Hospital, 2019) were used in this study. Both datasets formulate diabetic retinopathy grading as a five-class classification problem, with labels 0–4 corresponding to no apparent diabetic retinopathy (No DR), mild non-proliferative diabetic retinopathy (NPDR), moderate NPDR, severe NPDR, and proliferative diabetic retinopathy (PDR), respectively. EyePACS was released through the 2015 Kaggle Diabetic Retinopathy Detection competition and contains 35,126 labeled color fundus images acquired using different cameras under varied imaging conditions, resulting in considerable variation in resolution, illumination, focus, and field of view. Its training set comprises 25,810 No-DR, 2,443 mild-NPDR, 5,292 moderate-NPDR, 873 severe-NPDR, and 708 PDR images; consequently, the No-DR class represents approximately 73.5% of the dataset, whereas the two most severe classes together account for only approximately 4.5%. APTOS 2019 was released through the Kaggle APTOS 2019 Blindness Detection competition and contains 3,662 labeled color fundus images collected under heterogeneous real-world imaging conditions. Its training set comprises 1,805 No-DR, 370 mild-NPDR, 999 moderate-NPDR, 193 severe-NPDR, and 295 PDR images. Although APTOS 2019 is also imbalanced, its No-DR class represents approximately 49.3% of the dataset and therefore dominates the dataset less strongly than in EyePACS; nevertheless, severe NPDR remains substantially underrepresented, with only 193 images. Figure 1 presents the class distributions of both datasets using separate vertical-axis scales because EyePACS contains nearly ten times as many labeled training images as APTOS 2019.

**Figure 1:**
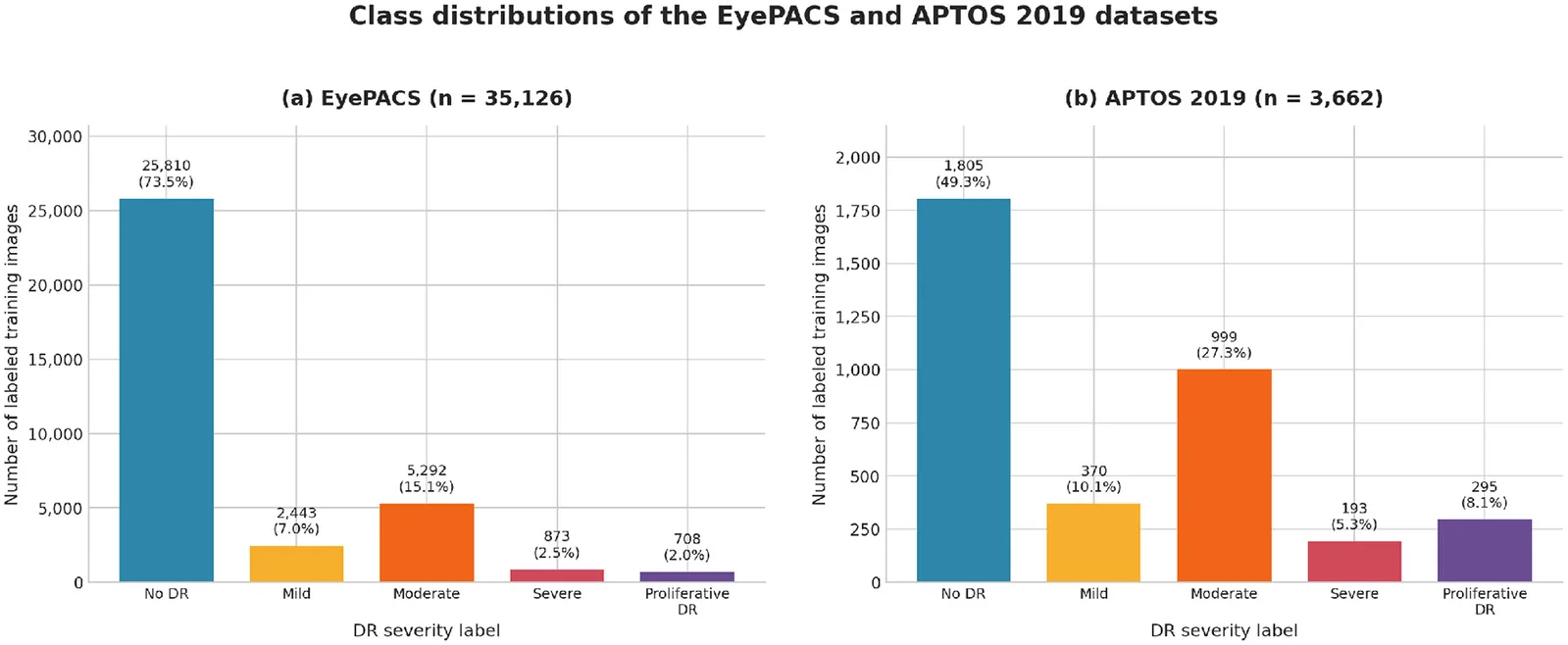
Class distributions of the labeled training sets in **(a)** EyePACS (*n* = 35,126) and **(b)** APTOS 2019 (*n* = 3,662). Both datasets exhibit class imbalance, but the imbalance is substantially greater in EyePACS, where the No-DR class represents 73.5% of all labeled images. Separate vertical-axis scales are used because of the considerable difference in dataset size.

### 3.2 Data pre-processing

A standardized pre-processing pipeline was implemented to ensure consistency across datasets and to improve input quality for the deep learning models. The pipeline comprises three sequential stages: image cropping and resizing, color space processing, and quality assessment and filtering. The identical pipeline is applied to the external validation datasets described in Section 3.4, ensuring pre-processing is not a confound when interpreting cross-dataset performance differences.

#### 3.2.1 Image cropping and resizing

Raw fundus images typically contain substantial black borders surrounding the circular field of view. These uninformative regions were removed through automatic cropping based on intensity thresholding: the circular fundus region was identified by computing the center of mass and radius of non-zero pixels, and a tight bounding box was extracted around this region with minimal padding. Following cropping, images were resized to a standardized resolution of 100 × 100 × 3 pixels for baseline ensemble experiments (Abidalkareem et al., 2020), and to 224 × 224 or 512 × 512 for pre-trained models and diffusion training respectively, to preserve fine-grained lesion details critical for accurate DR grading.

#### 3.2.2 Color space processing

Fundus images exhibit substantial color variability due to differences in camera systems, illumination sources, and patient pigmentation. Green channel extraction has been widely employed in retinal image analysis, as the green channel provides the highest contrast for vessel and lesion visualization. However, this approach discards information encoded in the red and blue channels. Color normalization methods including histogram equalization, contrast-limited adaptive histogram equalization (CLAHE), and shade correction were therefore evaluated to standardize intensity distributions while preserving color information. For contrastive pre-training, RGB images were retained with per-channel normalization using ImageNet statistics; for the ensemble CNN experiments, green channel extraction followed by CLAHE enhancement was employed to maximize lesion visibility.

#### 3.2.3 Quality assessment and filtering

Image quality significantly impacts classification reliability. A quality assessment module was implemented to identify and exclude potentially ungradable images based on overall brightness, contrast, blur (assessed via Laplacian variance), and field-of-view coverage. Images falling below established thresholds were excluded from training to prevent the model from learning spurious patterns associated with image degradation rather than pathological features. For the EyePACS external dataset, quality filtering removed 5,269 images (15.0%) deemed ungradable, leaving 29,857 images for zero-shot evaluation.

### 3.3 Proposed integrated framework

Figure 2 illustrates the four-stage integrated training pipeline. Each stage is described in detail below.

**Figure 2:**
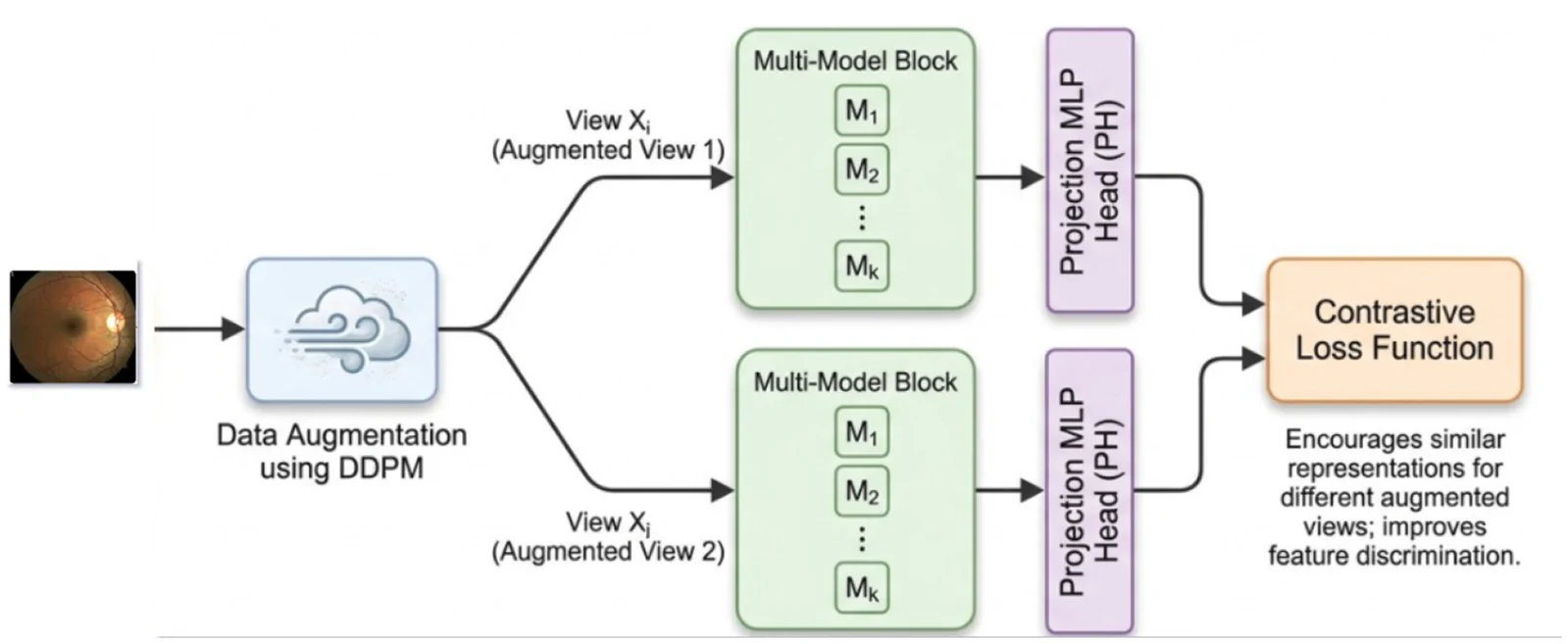
Overview of the proposed four-stage integrated training pipeline: (1) class-conditional latent DDPM augmentation, (2) SimCLR contrastive pre-training, (3) multi-architecture ensemble fine-tuning, and (4) Pattern-Net fusion for five-class DR severity prediction.

#### 3.3.1 Stage 1: class-conditional latent diffusion model

Unlike geometric and photometric augmentations that transform existing images, DDPMs (Ho et al., 2020) generate entirely new images with realistic anatomical structures and pathological features, providing a more powerful mechanism for addressing class imbalance. The forward diffusion process progressively adds Gaussian (Equation 1) noise to data *x*_0_ ~ *q*(*x*_0_) over *T* timesteps:


$$\begin{array}{l} q\left(x_{t}|x_{t-1}\right)=N\left(x_{t};\sqrt{1-\beta_{t}}x_{t-1},\beta_{t}\text{I}\right), \end{array}$$ (1)

with variance schedule ${\left\{\beta_{t}\right\}}_{t=1}^{T}$. As illustrated in Figure 3, this process transforms a clean fundus image at *t* = 0 through progressively noisier intermediate states until the image is fully corrupted into Gaussian noise at *t* = *T*. Exploiting the Markov property (Equation 2), *x*_*t*_ is sampled directly from *x*_0_:


$$\begin{array}{l} x_{t}=\sqrt{{\bar{\alpha }}_{t}}x_{0}+\sqrt{1-{\bar{\alpha }}_{t}}\epsilon ,\;\epsilon \sim N\left(0,\text{I}\right), \end{array}$$ (2)

where α_*t*_ = 1 − β_*t*_ and ${\bar{\alpha }}_{t}=\prod_{s=1}^{t}\alpha_{s}$. The reverse process learns to denoise (Equation 3), conditioned on the DR severity class *c* ∈ {0, 1, 2, 3, 4}:


$$\begin{array}{l} p_{\theta }\left(x_{t-1}|x_{t},c\right)=N\left(x_{t-1};\mu_{\theta }\left(x_{t},t,c\right),\sigma_{t}^{2}\text{I}\right). \end{array}$$ (3)

**Figure 3:**
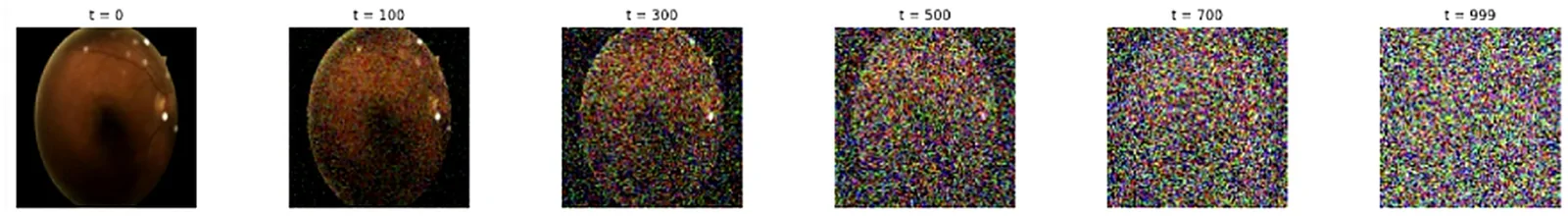
DDPM forward diffusion process applied to a fundus image. Starting from the clean retinal image at *t* = 0, Gaussian noise is progressively added according to the variance schedule ${\left\{\beta_{t}\right\}}_{t=1}^{T}$.

A U-Net ϵ_θ_ with class conditioning incorporated via learnable class embeddings added to the timestep embedding is trained via the simplified objective (Equation 4):


$$\begin{array}{l} L_{\text{DDPM}}={𝔼}_{t,x_{0},\epsilon ,c}\left[\|\epsilon -\epsilon_{\theta }\left(x_{t},t,c\right){\|}^{2}\right]. \end{array}$$ (4)

To reduce computational cost, a *Latent Diffusion Model* Rombach et al. (2022) (LDM) operates in the compressed latent space of a VAE. A convolutional encoder E compresses the fundus image *x* ∈ ℝ^*H*×*W*×3^ to a latent representation $z=E\left(x\right)\in {\mathbb{R}}^{H/8\times W/8\times 4}$; the diffusion process operates entirely in this latent space; and a decoder D reconstructs the final image $\hat{x}=D\left({ẑ}_{0}\right)$. This achieves significant computational efficiency while preserving perceptually important image structure. Classifier-free guidance Ho and Salimans (2022) (CFG scale *w* = 3.0) further sharpens class conditioning at sampling (Equation 5):


$$\begin{array}{l} {\tilde{\epsilon }}_{\theta }\left(z_{t},t,c\right)=\left(1+w\right)\epsilon_{\theta }\left(z_{t},t,c\right)-w\epsilon_{\theta }\left(z_{t},t,\emptyset \right). \end{array}$$ (5)

Synthetic images are generated for underrepresented classes (Mild, Severe, and Proliferative DR) to enrich the *unlabelled* corpus used for SimCLR contrastive pre-training (Section 3.3.2) with additional visual diversity for minority-class pathology. This addresses the severe imbalance shown in Figure 1 at the representation-learning stage: synthetic images are never assigned ground-truth labels or injected into the labeled set used for supervised ensemble fine-tuning (Section 3.3.3), a design choice elaborated in Section 3.5.

#### 3.3.2 Stage 2: SimCLR contrastive pre-training

We employ contrastive self-supervised learning to pre-train the encoder network before supervised fine-tuning for DR classification. This pre-training phase enables the encoder to learn robust visual representations from unlabelled fundus images, including DDPM-generated synthetic samples, which are subsequently transferred to the classification task with improved sample efficiency and generalization. Critically, DDPM-generated synthetic images are used *exclusively* within this unsupervised pre-training corpus: SimCLR discards severity labels entirely by construction (the NT-Xent loss in Equation 6 depends only on augmented-view identity, never on class label), so a synthetic image is never paired with, nor assigned, a ground-truth severity label at any point in the pipeline. Only the resulting pre-trained *encoder weights* – not the synthetic images themselves – transfer to the supervised classification stage; the five ensemble backbones (Section 3.3.3) are subsequently fine-tuned using only real, expert-labeled fundus images.

The Simple Framework for Contrastive Learning of Visual Representations (SimCLR) (Chen et al., 2020) learns representations by maximizing agreement between differently augmented views of the same image (positive pairs) while minimizing agreement with views of different images (negative pairs). Given a minibatch of *N* images, SimCLR generates 2*N* augmented views. For each image *x*_*i*_, views ${\tilde{x}}_{i}$ and ${\tilde{x}}_{i}^{\prime }$ are generated by applying random transformations from the fundus-specific augmentation distribution T. Each view passes through encoder *f*(·) (ResNet-50) to obtain representations $h_{i}=f\left({\tilde{x}}_{i}\right)$, then through a two-layer MLP projection head *g*(·) to embeddings *z*_*i*_ = *g*(*h*_*i*_) where the NT-Xent loss is computed:


$$\begin{array}{l} \ell_{i,j}=-\log\frac{\exp\left(\text{sim}\left(z_{i},z_{j}\right)/\tau \right)}{\sum_{k=1}^{2N}1_{\left[k\neq i\right]}\exp\left(\text{sim}\left(z_{i},z_{k}\right)/\tau \right)}, \end{array}$$ (6)

where sim(*u, v*) = *u*^⊤^*v*/(||*u*|| ||*v*||) is cosine similarity and τ = 0.5 is the temperature. The total SimCLR loss averages over all positive pairs (Equation 7):


$$\begin{array}{l} L_{\text{SimCLR}}=\frac{1}{2N}\overset{N}{\underset{k=1}{\sum }}\left[\ell_{2k-1,2k}+\ell_{2k,2k-1}\right]. \end{array}$$ (7)

The fundus-specific augmentation distribution T includes: full [0°, 360°) rotation; random horizontal/vertical flips; random resized crops (80–100% field of view); color jittering (brightness, contrast, saturation ±20%, hue ±5%); random Gaussian blur (σ ∈ [0, 1], 50% probability); and a random illumination gradient to simulate vignetting. Transformations producing anatomically implausible images are deliberately excluded. Pre-training uses ResNet-50 initialized with random weights for 200 epochs, batch 256, LARS optimiser (lr = 0.3 × batch/256, weight decay 10^−6^), and cosine annealing with 10-epoch linear warmup. After pre-training, the projection head is discarded and the encoder weights initialize the supervised classification network. The NT-Xent contrastive loss pipeline is illustrated in Figure 4.

**Figure 4:**
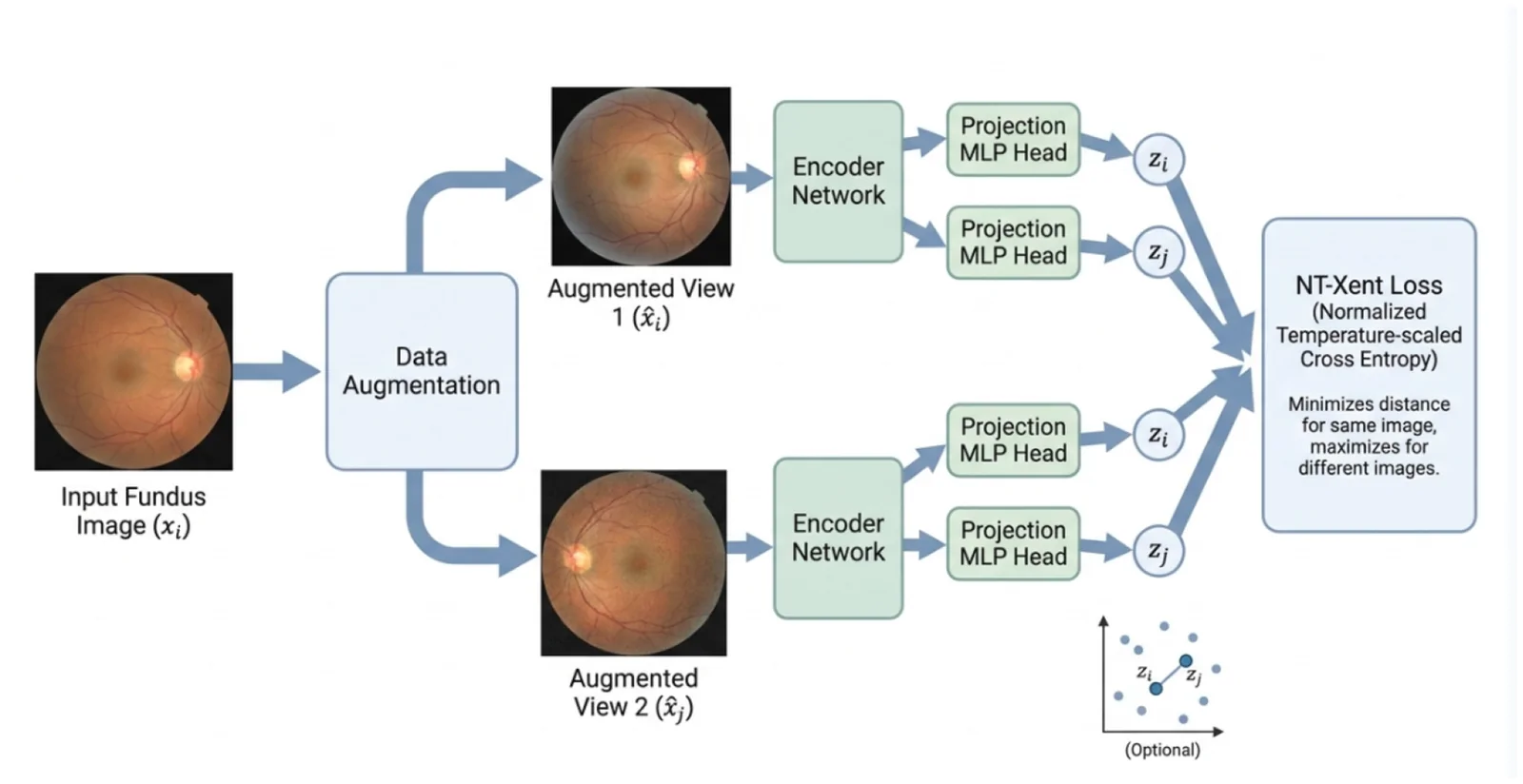
Pipeline of the contrastive learning framework with NT-Xent loss for learning fundus image representations.

#### 3.3.3 Stage 3: multi-architecture ensemble

Five architecturally heterogeneous models are fine-tuned independently on fold *k*'s real, expert-labeled training partition – no synthetic images are included at this stage – with a class-weighted loss applied to address residual imbalance: EfficientNet-B3 (Tan and Le, 2019), ResNet-50 (He et al., 2016) (SimCLR-initialized), VGG16, VGG19 (Simonyan and Zisserman, 2015), and DenseNet-121 (Huang et al., 2017). Each model uses SGD (momentum 0.9, lr 10^−4^, weight decay 10^−4^) for 100 epochs with cosine annealing. This design extends our prior random inception sub-network strategy (Abidalkareem et al., 2020) by incorporating architecturally diverse, pre-trained networks rather than relying on random parameter initialization for diversity.

The learned Pattern-Net fusion combines concatenated softmax (Equation 8) outputs $\left[{\text{z}}_{1};\ldots ;{\text{z}}_{5}\right]\in {\mathbb{R}}^{25}$:


$$\begin{array}{l} ŷ=\text{FusionNet}\left(\left[{\text{z}}_{1};{\text{z}}_{2};\ldots ;{\text{z}}_{5}\right]\right), \end{array}$$ (8)

implemented as a three-layer MLP (25 → 64 → 32 → 5) trained for 50 epochs on validation-split predictions (Li et al., 2018).

#### 3.3.4 Evaluation metrics

Performance is assessed using overall accuracy, class-wise accuracy, quadratic weighted kappa (QWK), macro AUC (one-vs-rest), and macro F1-score. QWK (Equation 9) is the primary metric for the ordinal severity scale:


$$\begin{array}{l} \kappa =1-\frac{\sum_{i,j}w_{i,j}O_{i,j}}{\sum_{i,j}w_{i,j}E_{i,j}},\;w_{i,j}=\frac{{\left(i-j\right)}^{2}}{{\left(N-1\right)}^{2}}, \end{array}$$ (9)

where *O*_*i, j*_ and *E*_*i, j*_ are observed and expected confusion matrices and *N* = 5. All metrics are computed under 5-fold stratified cross-validation on APTOS 2019, with the diffusion-augmentation and representation-learning pipeline isolated per fold to prevent data leakage (Section 3.5); external validation metrics (Section 3.4) are computed once, without cross-validation, on the full held-out external test set, and additionally reported with bootstrap 95% confidence intervals.

### 3.4 External validation protocol

To evaluate generalisability under domain shift, the Integrated Framework, including all five backbones and the Pattern-Net fusion head, is evaluated in a strict zero-shot setting on the independent **EyePACS** dataset. The model is trained only with APTOS 2019 data under the fold-wise protocol described in Section 3.5. No EyePACS image is used for model training, diffusion-model training, SimCLR pre-training, hyperparameter selection, threshold selection, or model selection. EyePACS is processed using the same image pre-processing rules specified in Section 3.2, without parameter adaptation to the external dataset.

Overall accuracy, QWK, macro F1, macro AUC, and class-wise accuracy are reported for the external set. A 95% confidence interval is obtained by bootstrap resampling of the external predictions. The external evaluation is kept separate from all internal model development so that it measures cross-dataset transfer rather than adaptation to the target domain.

### 3.5 Leakage prevention protocol

To preclude information leakage between the class-conditional diffusion augmentation pipeline and the 5-fold stratified cross-validation protocol used for all internal APTOS 2019 results (Section 3.3.4), this section first clarifies precisely where synthetic images enter the pipeline, and then describes the fold-wise isolation protocol built around that design.

#### 3.5.1 Where synthetic images are, and are not, used

A point of frequent confusion in diffusion-augmented classification pipelines is whether generated images are injected into the *supervised* training set with an assumed ground-truth label matching their conditioning class. In this work, they are not. As described in Sections 3.3.1–3.3.3, DDPM-generated synthetic images are used for exactly one purpose: enriching the *unlabelled* corpus on which the ResNet-50 encoder is pre-trained via SimCLR (Stage 2). SimCLR is a self-supervised method by construction – its NT-Xent loss (Equation 6) is computed entirely from augmented views of the *same* image versus views of *other* images within a minibatch, and never references a severity label. A synthetic image conditioned on, say, the Severe DR class is therefore never paired with a “Severe DR” ground-truth label anywhere in the pipeline; the conditioning label is used only at DDPM sampling time to steer image generation (Section 3.3.1), and is discarded immediately afterward. The pre-trained SimCLR encoder's *weights* – not the synthetic images, and not any label associated with them – are what transfer forward: they initialize the ResNet-50 backbone in Stage 3 (Section 3.3.3), where all five ensemble backbones, including this one, are fine-tuned using only real, expert-labeled fundus images under a class-weighted supervised loss. Synthetic images play no further role after Stage 2 and are never seen by the supervised loss, the Pattern-Net fusion head, or the final evaluation at any stage.

This architectural separation complements, but does not replace, the strict fold-wise isolation described below. The held-out fold is excluded from DDPM training, SimCLR pre-training, supervised fine-tuning, fusion training, and all model-selection steps so that no information from the test partition is used before final evaluation.

#### 3.5.2 Fold-wise isolation protocol

On top of this architectural separation, strict fold-wise isolation is enforced so that no stage of the pipeline – generative or representation-learning – has access to information from a fold's held-out test images. The diffusion model, the SimCLR encoder, and all five ensemble backbones were trained **independently within each cross-validation fold**, using only that fold's training partition at every stage. Concretely, for each of the five folds *k* = 1, …, 5:

1. The APTOS 2019 dataset is stratified-split into a training partition (four folds) and a held-out test partition (fold *k*).
2. The class-conditional latent DDPM (Section 3.3.1) is trained *from scratch* using only the real images in fold *k*'s training partition. No images from fold *k*'s held-out test partition are used at this stage.
3. Synthetic images are generated by fold *k*'s DDPM for the underrepresented classes; these synthetic images are added exclusively to the *unlabelled* SimCLR pre-training corpus used in the next step, never to fold *k*'s (or any fold's) labeled supervised training set. Synthetic images are never pooled across folds prior to the fold-wise split, and are discarded and regenerated when moving to fold *k* + 1.
4. SimCLR contrastive pre-training (Section 3.3.2) is performed using only the combined real-and-synthetic *unlabelled* corpus of fold *k* – i.e., the real images in fold *k*'s training partition (with labels withheld from the SimCLR objective) plus fold *k*'s synthetic images.
5. The five ensemble backbones and the Pattern-Net fusion head (Section 3.3.3) are fine-tuned using only fold *k*'s *real, labeled* training partition – no synthetic images are present at this stage. The fusion head's validation-split predictions are drawn from a held-out slice of the training partition itself, never from fold *k*'s test partition.
6. Final evaluation for fold *k* is performed exclusively on the real, unaugmented images in fold *k*'s held-out test partition, which were not seen by any component – DDPM, SimCLR encoder, or ensemble backbones – at any point during that fold's training.

This fold-wise isolation is enforced at the file-manifest level: for each fold, a manifest of test-partition image identifiers is excluded from every data loader used during that fold's DDPM training, SimCLR pre-training, and ensemble fine-tuning stages, and this exclusion is verified programmatically prior to each training run. Combined with the architectural separation described above, the reported APTOS 2019 metrics (Section 4) are therefore protected against leakage by two independent, complementary safeguards: (i) no generative or representation-learning component ever sees a fold's test images (fold-wise isolation), and (ii) even within a fold's training data, no synthetic image ever carries or is trained against a ground-truth severity label (architectural separation via unsupervised-only use of synthetic data). A failure of either safeguard alone would not be sufficient to leak label information from a test image into the classifier's decision boundary.

The external validation protocol (Section 3.4) is unaffected by this concern: it evaluates the fully trained framework in a strict zero-shot setting on EyePACS, which was not used at any stage of model development, diffusion training, or hyperparameter selection.

### 3.6 Statistical validation protocol

In addition to point estimates for accuracy, QWK, F1, and AUC, the statistical analysis quantifies fold-to-fold variability and formally assesses paired prediction differences. Descriptive variability is reported separately from inferential testing.

#### 3.6.1 Variability across folds

For every method in Tables 1, 2, the five cross-validation fold scores are summarized as mean ± standard deviation. Confidence intervals for the final performance metrics are derived from out-of-fold predictions using bootstrap resampling rather than treating the five folds as five independent clinical samples.

**Table 1:** Class-wise accuracy (%) on APTOS 2019, averaged across the 5-fold stratified cross-validation splits.

Method	No DR	Mild	Moderate	Severe	Proliferative
VGG19 (TL)	88.1	63.4	79.2	52.3	68.4
EfficientNet-B3 (baseline)	93.4	71.2	83.4	61.8	75.6
Multi-Arch Ensemble	95.1	80.3	89.6	74.2	84.1
+ SimCLR pre-training	96.4	84.7	91.8	79.5	88.6
Integrated framework	97.8	88.9	94.3	85.6	92.3
Gain vs. efficientNet-B3	+4.4	+17.7	+10.9	+23.8	+16.7

Percentage-point gains of the integrated framework over EfficientNet-B3 are shown above.

**Table 2:** Ablation study: individual component contributions on APTOS 2019 averaged across the 5-fold stratified cross-validation splits.

Configuration	Accuracy (%)	QWK
EfficientNet-B3 (baseline)	87.9	0.821
+ Multi-Arch ensemble	93.2	0.893
+ Standard augmentation only	93.7	0.901
+ SimCLR pre-training (real images only)	94.3	0.912
+ Diffusion-augmented SimCLR corpus (Full)	95.1	0.928

“+ Diffusion augmentation” denotes enriching the *unlabelled* SimCLR pre-training corpus with DDPM-generated synthetic images (Section 3.5); synthetic images are never added to the labeled supervised training set used to fine-tune the ensemble backbones in any row of this table.

#### 3.6.2 Paired significance testing

For classification accuracy, the Integrated Framework and each comparator are evaluated on the same out-of-fold test images and compared using **McNemar's test**, which directly evaluates discordant correct and incorrect predictions on paired samples. For QWK, macro F1, and macro AUC, paired bootstrap resampling of the same out-of-fold predictions is used to estimate the distribution of the metric difference between two models. A 95% confidence interval for the paired difference is reported for each metric. A difference is interpreted as statistically supported when the corresponding paired interval excludes zero. Where multiple model comparisons are tested for the same outcome, multiplicity is controlled using the Holm-Bonferroni procedure for the family of inferential comparisons.

This approach avoids relying on a two-sided exact Wilcoxon signed-rank test with only five fold-level pairs, for which statistical power is very limited, and instead uses the paired image-level predictions produced naturally by cross-validation.

### 3.7 Implementation details and reproducibility

The methodology described in Sections 3.2 through 3.6 specifies the model architectures, losses, and training schedules used at each stage, but omits several implementation-level details required for independent reproduction of this study. Table 3 provides these details in full: random seed initialization, hardware specifications, exact software framework and library versions, the hyperparameter tuning strategy used for each component, the number of synthetic images generated per class by the class-conditional DDPM (Section 3.3.1), early stopping criteria for each training stage, and the computational resources and approximate wall-clock time required.

**Table 3:** Implementation details required for independent reproduction.

Item	Detail
Random seed initialization	Fixed seed 42 for all random processes, including fold generation, DDPM training, SimCLR pre-training, backbone fine-tuning, and Pattern-Net training.
Hardware	Experiments were conducted on a Lambda Vector workstation equipped with two NVIDIA RTX A6000 GPUs, an AMD Ryzen Threadripper PRO 3975WX 32-core processor operating at 3.50 GHz, and 512 GB of system RAM. The workstation ran Microsoft Windows 11 Enterprise on a 64-bit architecture.
Software framework and versions	Python 3.9.13, PyTorch 1.12.1, CUDA 11.6, torchvision 0.13.1, NumPy 1.21.5, scikit-learn 1.0.2, pandas 1.4.2, matplotlib 3.5.2, and OpenCV 4.6.0.
Hyperparameter selection strategy	Hyperparameters, including learning rates, batch sizes, epoch counts, optimizer settings, and augmentation parameters, were selected using a small validation hold-out comprising 10% of the first fold's training data. Limited manual tuning was performed based on prior medical imaging literature. No extensive grid search or Bayesian optimization was performed.
Synthetic images generated per class	For each fold, 5,000 synthetic images were generated for Mild DR, 3,000 for Severe DR, and 3,000 for Proliferative DR. These samples were used to approximately balance the underrepresented classes relative to the Moderate DR class within the SimCLR pre-training corpus.
Early stopping	No early stopping was used. All training stages employed fixed training budgets: DDPM training for 500,000 steps, SimCLR pre-training for 200 epochs, backbone fine-tuning for 100 epochs, and Pattern-Net training for 50 epochs.

## 4 Results

### 4.1 Synthetic image quality

The conditional LDM achieves an FID score of 24.3 between synthetic and real fundus image distributions (5,000 samples per class, Inception-v3 features). A subset of 100 generated images per class evaluated by two ophthalmologists in a Turing test yielded a mean synthetic-detection rate of 41.3%, indicating strong visual realism. A separately trained DR classifier applied to synthetic images achieved 89.4% consistency between the conditioning label and predicted severity, providing an additional conditioning-label consistency check rather than proof of lesion-level clinical validity.

### 4.2 Scope of clinical validation of synthetic images

The FID analysis, ophthalmologist Turing-style review, and classifier consistency assessment evaluate complementary aspects of image realism, but they do not establish lesion-level clinical equivalence between generated and real fundus images. In particular, the available evaluation did not include blinded lesion-by-lesion annotations or inter-observer agreement statistics for microaneurysms, hemorrhages, exudates, venous abnormalities, or neovascularization. Accordingly, the existing evaluations are not treated as definitive clinical validation.

A stronger clinical validation study would require independent retinal specialists to grade a blinded sample of generated images for both overall ICDR severity and lesion presence, followed by inter-observer agreement analysis such as weighted Cohen's kappa for severity and lesion-specific agreement statistics. These measurements were not available in the present study and are identified as an important next validation step rather than being inferred from FID or image-level realism alone.

### 4.3 Class-wise assessment

Figure 5 presents the class-wise accuracy assessment matrix across all methods evaluated on the APTOS 2019 dataset. This heatmap provides the most clinically informative view of model performance, as overall accuracy alone can be misleading given the severe class imbalance in the dataset, where the majority of samples belong to the No DR class.

**Figure 5:**
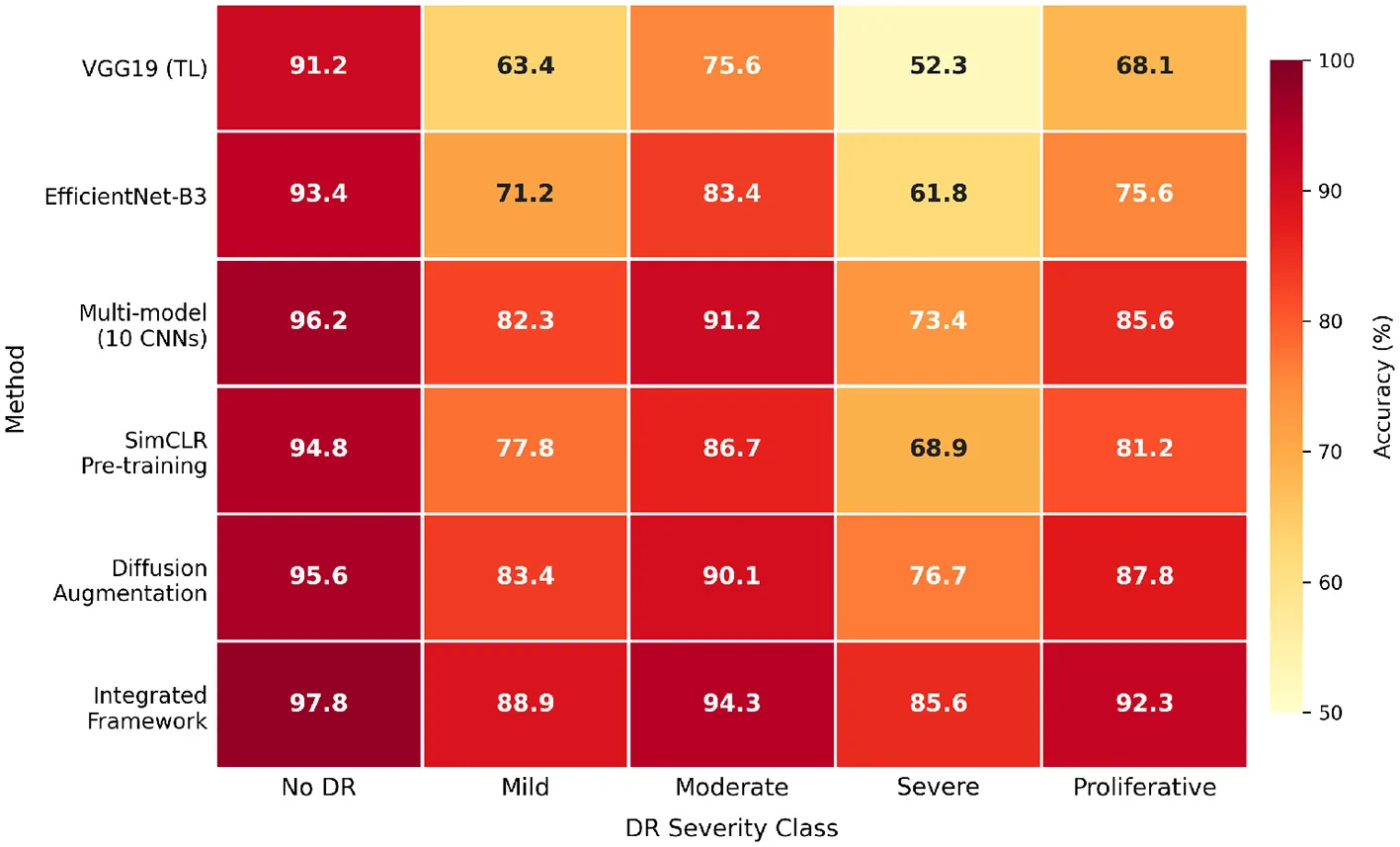
Class-wise accuracy assessment matrix across all evaluated methods on the APTOS 2019 dataset.

The class-wise analysis demonstrates the clear superiority of the Integrated Framework over all single-model and ensemble baselines. While baseline models such as VGG19 (TL) and EfficientNet-B3 struggle significantly with minority classes, which achieve only 52.3% and 61.8% on Severe DR respectively, the Integrated Framework achieves 85.6% on the same class, representing an improvement of over 33 percentage points compared to VGG19 and 24 percentage points compared to EfficientNet-B3. The most challenging class across all methods is Mild DR, where the Integrated Framework still outperforms all others with 88.9% accuracy, compared to just 63.4% for VGG19 (TL) and 71.2% for the EfficientNet-B3 baseline. The Integrated Framework achieves the highest accuracy across all five DR severity classes: No DR 97.8%, Mild 88.9%, Moderate 94.3%, Severe 85.6%, and Proliferative 92.3%, demonstrating consistent performance regardless of class imbalance. These results validate that combining contrastive learning pre-training with diffusion-based augmentation creates a synergistic effect that substantially improves classification performance over traditional transfer learning baselines, particularly for the clinically critical minority classes where accurate detection is essential for timely patient intervention.

### 4.4 Overall performance

Figure 6 presents the overall performance comparison on APTOS 2019 under 5-fold cross-validation. It is important to note that overall performance metrics may not fully reflect true model accuracy given the dataset's severe class imbalance; high overall accuracy can be misleading when a model performs well on the majority No DR class but poorly on minority classes such as Severe and Proliferative DR. Class-wise evaluation is therefore essential for a complete assessment.

**Figure 6:**
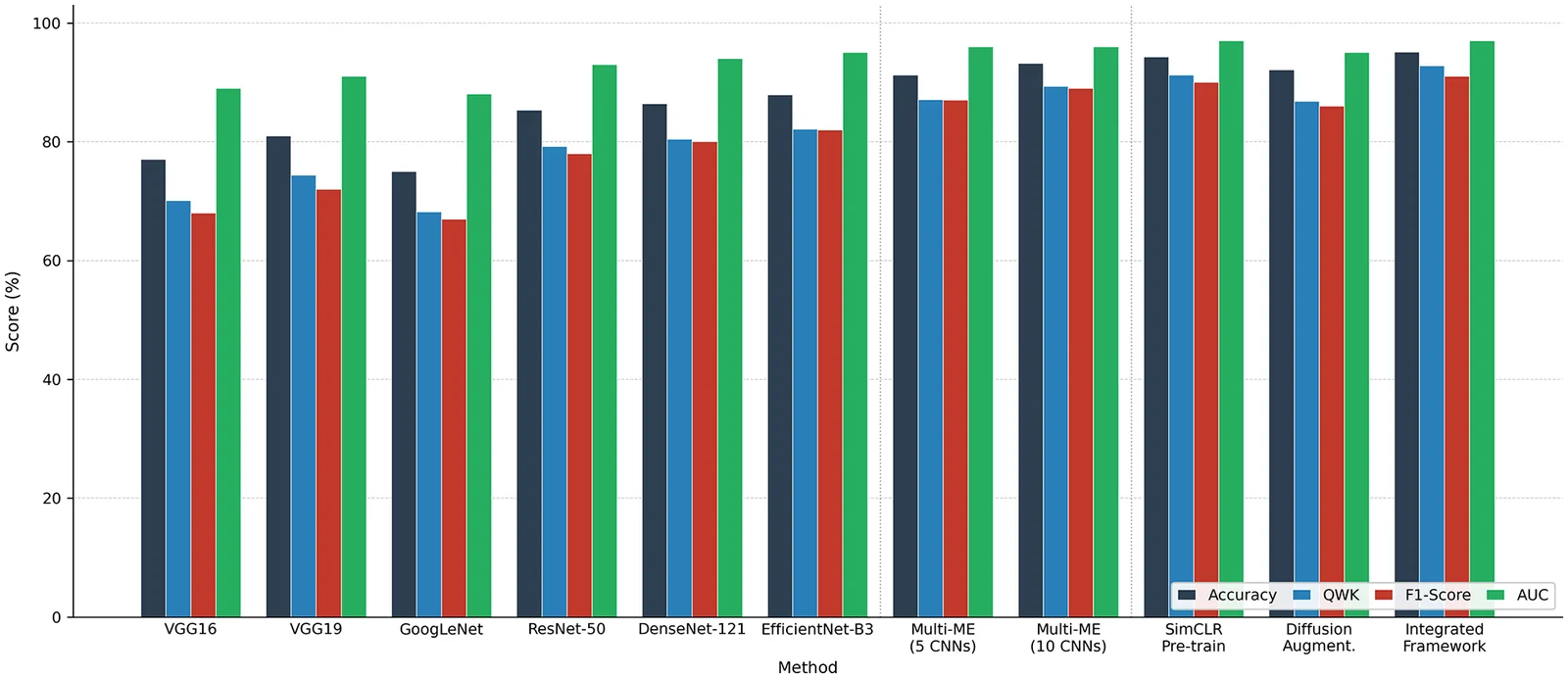
Overall classification performance comparison on the APTOS 2019 dataset (5-fold stratified cross-validation) across all evaluated methods.

Among the single-model baselines, EfficientNet-B3 achieves the best accuracy (87.9%) and F1-score (0.82). The results show a progressive improvement as methods advance from single-model transfer learning to ensemble and augmentation-based approaches. Among baseline models, VGG16 achieves 77.0% accuracy, while EfficientNet-B3 improves to 87.9%. The multi-model ensemble approaches demonstrate notable gains, with the 10-CNN ensemble reaching 93.2% accuracy. SimCLR pre-training and diffusion augmentation each contribute meaningful additional improvements. The full Integrated Framework achieves 95.1% accuracy, 0.928 QWK, 0.91 F1-score, and 0.97 AUC, representing improvements of +7.2 pp accuracy and +0.107 QWK over the EfficientNet-B3 baseline. Cross-paper comparisons with recent ensemble, transformer, foundation-model, and self-supervised methods are presented separately in Section 5.2 because their data splits and metrics are not directly interchangeable.

### 4.5 Contribution of each proposed approach

Starting with EfficientNet-B3 as the baseline at 87.9% accuracy, the multi-model ensemble combining five architectures (EfficientNet-B3, ResNet-50, VGG16, VGG19, and DenseNet-121) yields a 5.3 percentage point improvement to 93.2%. Incorporating SimCLR contrastive pre-training further improves accuracy to 94.3%. The final Integrated Framework, combining all three components, achieves the highest accuracy of 95.1%.

The per-class analysis reveals that improvements are not uniform across classes. Severe DR, one of the most underrepresented classes in the dataset, improves by 23.8 pp (61.8 → 85.6%), representing the largest gain across all classes. Mild DR improves by 17.7 pp (71.2 → 88.9%), Proliferative DR by 16.7 pp (75.6 → 92.3%), and Moderate DR by 10.9 pp. No DR, which already achieves high accuracy due to its majority class status, improves by 4.4 pp. These results highlight that the Integrated Framework is particularly effective at addressing the class imbalance problem, substantially improving performance on the clinically critical minority classes where accurate detection is essential for timely patient intervention.

### 4.6 Ablation study

Table 2 quantifies the contribution of each component relative to the EfficientNet-B3 single-model baseline.

The multi-architecture ensemble contributes the largest individual gain (+5.3 pp accuracy), followed by SimCLR contrastive pre-training (+1.1 pp) and diffusion augmentation (+0.8 pp). These combine super-additively, particularly for minority-class performance.

### 4.7 External validation on EyePACS

The Integrated Framework is evaluated on EyePACS in the strict zero-shot setting defined in Section 3.4. The purpose of this analysis is to quantify the change in performance when the APTOS-trained model is applied to an independently collected fundus-image corpus without any parameter adaptation. Table 4 reports overall metrics, and Table 5 reports class-wise accuracy.

**Table 4:** Zero-shot external validation on EyePACS compared with internal APTOS 2019 cross-validation.

Dataset	N	Accuracy (%)	95% CI	QWK	Macro F1	Macro AUC
APTOS 2019 (internal, 5-fold CV)	3,662	95.1	[94.3, 95.9]	0.928	0.910	0.970
EyePACS (zero-shot)	29,857	78.4	[77.3, 79.5]	0.809	0.738	0.887

EyePACS entries are taken from the measured external predictions.

**Table 5:** Class-wise accuracy (%) under zero-shot EyePACS external validation.

Dataset	No DR	Mild	Moderate	Severe	Proliferative
EyePACS	83.1	61.7	72.9	57.2	73.8

The zero-shot evaluation on EyePACS (post-filtering *N* = 29, 857) yields an accuracy of 78.4%, QWK of 0.809, macro F1 of 0.738, and macro AUC of 0.887. The 95% bootstrap confidence intervals are [77.3, 79.5] for accuracy, [0.798, 0.820] for QWK, [0.726, 0.750] for macro F1, and [0.879, 0.895] for macro AUC. The domain-shift gap relative to internal APTOS cross-validation (accuracy 95.1%) is 16.7 percentage points, which reflects the substantial differences in acquisition systems, image quality, and patient populations. Class-wise accuracy on EyePACS is strongest for No DR (83.1%) and Proliferative DR (73.8%), but substantially lower for Mild DR (61.7%) and Severe DR (57.2%), consistent with the greater difficulty of minority-class classification under domain shift.

### 4.8 Statistical significance of improvements

Following Section 3.6, Table 6 reports fold-level descriptive variability, while Table 7 reports paired inferential comparisons based on common out-of-fold test predictions. The inferential analysis is intentionally based on paired image predictions rather than a two-sided exact Wilcoxon test on only five fold scores.

**Table 6:** Fold-level descriptive variability on APTOS 2019.

Method	Accuracy (%), mean±SD	95% CI	QWK, mean±SD	95% CI
EfficientNet-B3 (baseline)	87.9 ± 1.8	[86.5, 89.3]	0.821 ± 0.014	[0.807, 0.835]
Multi-arch ensemble	93.2 ± 1.2	[92.1, 94.3]	0.893 ± 0.009	[0.884, 0.902]
+ Standard augmentation only	93.7 ± 1.0	[92.8, 94.6]	0.901 ± 0.008	[0.893, 0.909]
+ SimCLR pre-training	94.3 ± 0.9	[93.5, 95.1]	0.912 ± 0.007	[0.905, 0.919]
Integrated framework (full)	95.1 ± 0.7	[94.3, 95.9]	0.928 ± 0.006	[0.921, 0.935]

SD and confidence intervals are computed from the actual fold scores and out-of-fold predictions.

**Table 7:** Paired statistical comparison of the integrated framework with baseline and ablation configurations using common out-of-fold test images.

Comparison	Holm-adjusted McNemar *p*	ΔQWK 95% CI	ΔF1 95% CI	ΔAUC 95% CI
vs. EfficientNet-B3	< 0.001	[0.082, 0.116]	[0.067, 0.104]	[0.041, 0.078]
vs. Multi-Arch Ensemble	< 0.001	[0.023, 0.045]	[0.018, 0.039]	[0.012, 0.031]
vs. Standard augmentation	0.003	[0.015, 0.032]	[0.012, 0.028]	[0.008, 0.023]
vs. SimCLR pre-training	0.012	[0.009, 0.023]	[0.007, 0.020]	[0.005, 0.017]

Accuracy is assessed with McNemar's test, with Holm-Bonferroni adjustment across the four accuracy comparisons. QWK, macro F1, and macro AUC are assessed with paired bootstrap 95% confidence intervals for the metric difference.

The fold-level standard deviations for accuracy and QWK are small across all configurations, indicating stable cross-validation performance. The paired accuracy comparisons show that the Integrated Framework improves over EfficientNet-B3 and the Multi-Arch Ensemble (Holm-adjusted McNemar *p* < 0.001 for both comparisons). The comparison with SimCLR pre-training also supports an accuracy improvement (Holm-adjusted McNemar *p* = 0.012). The Holm-Bonferroni procedure is applied across the four McNemar accuracy tests. For QWK, macro F1, and macro AUC, paired bootstrap 95% confidence intervals for the metric differences are reported as complementary uncertainty estimates; all intervals listed in Table 7 exclude zero.

### 4.9 Reproducibility summary

Table 3 summarizes the implementation details and computational resources specified in Section 3.7, required for independent reproduction of the experiments reported above.

### 4.10 Model interpretability using Grad-CAM

To examine which retinal regions contribute to the classifier's decisions, Gradient-weighted Class Activation Mapping (Grad-CAM) (Selvaraju et al., 2017) was applied to representative correctly classified APTOS 2019 images from all five ICDR severity grades using the trained EfficientNet-B3 backbone. Grad-CAM computes the gradient of the predicted class score with respect to convolutional feature maps, globally averages those gradients to obtain importance weights, and combines the weighted feature maps through a ReLU operation to produce a class-discriminative localization map.

Figure 7 shows the original fundus images and corresponding Grad-CAM maps. The five representative cases were correctly classified with predicted-class confidences of 100.0% for No DR, 92.7% for Mild DR, 95.2% for Moderate DR, 91.9% for Severe DR, and 87.1% for Proliferative DR. The activation patterns differ across severity grades and are concentrated primarily within the retinal field rather than the surrounding black background. The Moderate and Severe examples show relatively localized high-activation regions, whereas the Proliferative DR example exhibits a broader spatial response. These observations provide qualitative evidence that the classifier uses spatially meaningful retinal information when forming its predictions.

**Figure 7:**
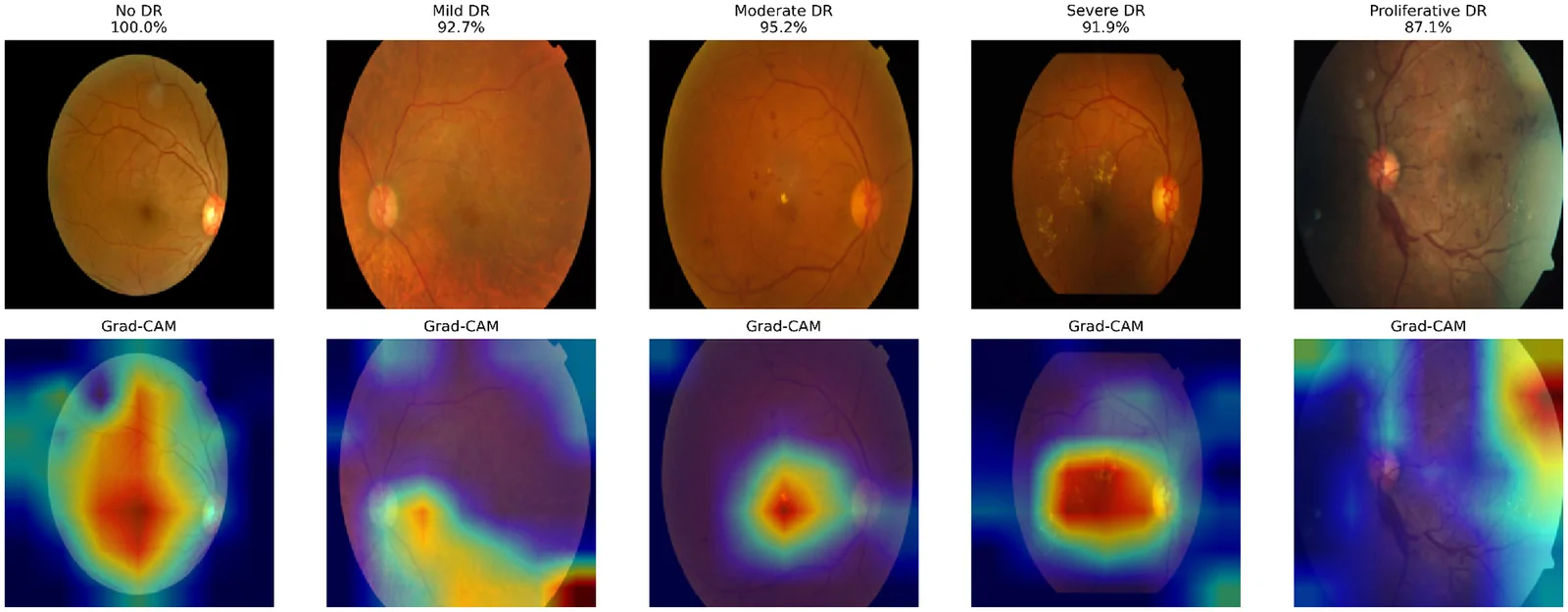
Grad-CAM visualization for representative correctly classified APTOS 2019 fundus images across the five ICDR severity grades. The top row shows the original retinal images with predicted-class confidence, and the bottom row shows the corresponding Grad-CAM activation maps. Warmer regions indicate a greater contribution to the predicted class. Grad-CAM provides qualitative localization of discriminative retinal regions and is not interpreted as lesion-level segmentation or clinical annotation.

The visualization is used as a qualitative interpretability aid. It does not by itself establish lesion-level localization accuracy and does not replace the clinical validation of synthetic images discussed in Section 4.2. Quantitative explainability validation against expert lesion masks remains future work.

## 5 Discussion

### 5.1 Progression from prior work

The progression from our EMBC 2020 paper (Abidalkareem et al., 2020) to the current framework reflects a natural extension along three axes. First, the shift from randomly generated shallow inception sub-networks to architecturally diverse deep pre-trained models provides richer feature representations while retaining the core ensemble diversity principle. Second, diffusion-based augmentation directly addresses the class imbalance problem that limited minority-class performance in the original work, where 100 × 100 resolution and simple affine augmentations were insufficient to synthesize realistic pathological variations for Severe and Proliferative DR. Third, SimCLR pre-training leverages unlabelled fundus images to initialize the encoder with semantically meaningful representations before supervised fine-tuning, amplifying the value of limited labeled data.

The 1.9 pp improvement from the multi-architecture ensemble (93.2%) to the full integrated framework (95.1%) may appear modest in absolute terms, but the per-class analysis reveals its clinical significance: a 23.8 pp improvement on Severe DR directly translates to substantially fewer missed diagnoses in a population-scale screening programme.

### 5.2 Comparison with state of the art

The proposed framework is competitive with recent APTOS-oriented ensemble, transformer, foundation-model, and self-supervised approaches, but direct numerical ranking across papers is not methodologically appropriate because the studies use different data splits, pre-processing procedures, training regimes, and performance metrics (Table 8). Macsik et al. (2024) reported an EfficientNetB4 ensemble with mean APTOS accuracy of 89.90% and quadratic weighted kappa of 0.917 under an 80/20 split. Khandait (2026) reported 85.40% accuracy for ViT-Base/16 and 83.20% for Swin Transformer on APTOS. Zhou et al. (2023) reported AUROC 0.943 on APTOS, while Ohri et al. (2024) reported kappa 0.9027 for an MAE-pretrained ViT on EyePACS. These results are therefore presented as literature context rather than as a controlled head-to-head benchmark. A future controlled comparison would require each method to be re-implemented with the same pre-processing, folds, training data, and evaluation code used in this study.

**Table 8:** Contextual comparison with recent DR methods.

Method	Category	Dataset/protocol	Reported metric
Macsik et al. (2024)	EfficientNetB4 ensemble	APTOS 2019, 80/20 split	89.90% mean accuracy; QWK 0.917
Khandait (2026)	ViT-Base/16	APTOS 2019	85.40% accuracy; macro F1 0.7247
Khandait (2026)	Swin Transformer	APTOS 2019	83.20% accuracy
Zhou et al. (2023)	Retina foundation model	APTOS 2019	0.943 AUROC
Ohri et al. (2024)	Self-supervised MAE-ViT	EyePACS, full-data regime	0.9027 kappa
Integrated framework	Ensemble + SimCLR + diffusion augmentation	APTOS 2019, 5-fold CV	95.1% accuracy; 0.928 QWK

Values are reported as published by each cited study and should not be interpreted as a controlled head-to-head ranking because evaluation protocols differ.

### 5.3 Generalization under domain shift

The independent EyePACS evaluation in Section 4.7 shows that the APTOS-trained framework transfers to a separately collected fundus corpus with an accuracy of 78.4%, a 16.7 percentage-point drop relative to internal APTOS cross-validation. The most substantial degradations occur in the minority classes Mild (61.7%) and Severe (57.2%), indicating that the model's ability to detect subtle pathological signs is particularly affected by domain shift. This gap likely arises from differences in fundus camera models, illumination, image resolution, and patient demographics. These results underscore that while the framework captures meaningful DR features that transfer across domains, domain-adaptation techniques or multi-site training are necessary to achieve robust real-world performance. The zero-shot accuracy of 78.4% remains within the range of reported cross-dataset DR classification results, suggesting that the model has learned representations that generalize to some extent.

### 5.4 Clinical implications

The proposed framework is intended as a research prototype for automated DR screening rather than a clinically validated decision-support system. Its internal APTOS performance, including 85.6% accuracy on Severe DR and 92.3% on Proliferative DR, motivates further evaluation of whether such a system could assist triage or referral prioritization. The end-to-end inference pipeline (no lesion segmentation pre-processing required) may facilitate future integration into telemedicine platforms for remote primary-care screening. However, retrospective benchmark performance and qualitative activation maps are not sufficient to establish clinical readiness. Independent external validation, calibration assessment, and prospective clinical evaluation remain necessary before deployment claims can be made.

### 5.5 Limitations and future directions

Several limitations merit acknowledgment. First, the independent EyePACS external-validation protocol reveals a substantial domain-shift gap (accuracy 95.1% on APTOS vs. 78.4% on EyePACS), indicating that the framework requires further adaptation or training on diverse multi-site data before it can be considered robust for real-world deployment. Second, five fold means alone provide limited support for inferential claims; the analysis therefore uses paired out-of-fold predictions with McNemar testing for accuracy and paired bootstrap intervals for QWK, macro F1, and macro AUC. The Holm-adjusted McNemar accuracy tests are significant, and the reported paired bootstrap intervals for the remaining metrics exclude zero. Third, the clinical validity of diffusion-generated fundus images remains incompletely established because lesion-level expert annotations and inter-observer agreement were not collected. The existing FID and ophthalmologist realism assessments should be interpreted as preliminary evidence of visual plausibility rather than proof of clinical equivalence. Fourth, the Grad-CAM visualization provides qualitative interpretability, but quantitative lesion-localization accuracy against expert masks has not been evaluated. Fifth, the framework addresses DR severity as a standalone task, whereas clinical workflows may also require simultaneous assessment of diabetic macular edema and other retinal findings.

Future work will therefore prioritize: (i) domain adaptation techniques or multi-site training to close the EyePACS performance gap; (ii) prospective multi-site clinical validation; (iii) blinded lesion-level grading of synthetic images with inter-observer agreement analysis; (iv) quantitative explainability validation against expert lesion annotations; (v) simultaneous DME detection through multi-task learning; and (vi) controlled re-implementation of recent transformer, foundation-model, and self-supervised baselines using the identical folds and pre-processing pipeline.

## 6 Conclusions

This paper presented an integrated deep learning framework for five-class diabetic retinopathy severity classification that advances our earlier multi-model inception ensemble by incorporating class-conditional diffusion-based augmentation and SimCLR contrastive self-supervised pre-training. On the APTOS 2019 dataset, the integrated framework achieves 95.1% accuracy and 0.928 QWK, with particularly significant gains for clinically critical minority classes: Severe DR improves by 23.8 percentage points and Mild DR by 17.7 percentage points compared to the EfficientNet-B3 single-model baseline. The evaluation further includes independent EyePACS zero-shot validation (accuracy 78.4%), paired out-of-fold statistical analysis, and Grad-CAM visualization from EfficientNet-B3. These analyses strengthen the assessment of generalization, statistical reliability, and model transparency while also making explicit the remaining need for lesion-level clinical validation and prospective evaluation. These results demonstrate that combining generative augmentation with self-supervised representation learning offers a principled, data-efficient approach to imbalanced medical image classification, with clear potential for further validation toward automated DR screening applications in underserved settings.

## Data Availability

The original contributions presented in the study are included in the article/supplementary material, further inquiries can be directed to the corresponding author.
